# Dentists’ Attitudes toward Diabetes Mellitus Screening in Thai Dental Clinics

**DOI:** 10.3390/ijerph19063341

**Published:** 2022-03-11

**Authors:** Chanita Tantipoj, Thaksaporn Sirichanyaphong, Jiratchaya Nuntachurat, Kriddichon Ruetaijetjaroen, Narin Hiransuthikul, Patr Pujarern, Pornpoj Fuangtharnthip, Siribang-on Piboonniyom Khovidhunkit

**Affiliations:** 1 Department of Advanced General Dentistry, Faculty of Dentistry, Mahidol University, Ratchathewi, Bangkok 10400, Thailand; chanita.tat@mahidol.edu (C.T.); patr.puj@mahidol.ac.th (P.P.); pornpoj.fun@mahidol.ac.th (P.F.); 2 Mahidol International Dental School, Faculty of Dentistry, Mahidol University, Ratchathewi, Bangkok 10400, Thailand; thaksaporn.sii@student.mahidol.ac.th (T.S.); jiratchaya.nut@student.mahidol.ac.th (J.N.); kriddichon.rut@student.mahidol.ac.th (K.R.); 3 Department of Preventive and Social Medicine, Faculty of Medicine, Chulalongkorn University, Bangkok 10330, Thailand; nhiransu@gmail.com

**Keywords:** type 2 diabetes mellitus, screening, attitude, questionnaires, dentists

## Abstract

Diabetes mellitus (DM), especially type 2 DM, has become a common problem worldwide. Previous studies have demonstrated that chairside screening can effectively identify undetected type 2 DM patients. This study was conducted to determine dentists’ attitudes toward DM screening in dental clinics. A total of 632 currently active dentists with more than 1 year of working experience participated. The six-part (importance, barriers, willingness, readiness, knowledge, and routine management) anonymous, self-administered questionnaire of five-point response scales was then distributed. The results illustrated that most dentists (86.3%) realized the importance of DM screening and that patients’ willingness was the main potential barrier (86.4%). Of the respondents, 98.1% and 82.4% were willing to measure blood pressure and weight and height for their patients, whereas only 45.4% and 38.8% were willing to collect blood from the fingertip or oral fluids for salivary diagnostics, respectively. Moreover, 73.7% of respondents were ready to refer patients to physicians, and 59.5% could explain the relationship between DM and oral diseases. However, only 44.3% and 27.9% were prepared to provide education about DM awareness or were able to perform screening, respectively. In addition, 67.2% and 65.8% knew the screening criteria and risk factors of DM, respectively, but only 45.1% knew what to do. The result of our study provided essential knowledge with respect to dentists’ attitudes in the screening for DM in Thai dental clinics.

## 1. Introduction

From the oral professional’s point of view, having proper oral health has been considered an essential portion of the individuals’ overall health. This key has been supported by the relationship between oral health and systemic health [1,2]. Diabetes mellitus (DM), in particular type 2 DM, is considered as one of the diseases playing a crucial part in the oral health condition and is known to correlate with periodontitis [3]. Hyperglycemia due to DM negatively influences oral health, whereas severe periodontitis can also negatively affect glycemic control. Therefore, the relationship between DM and periodontitis is considered bidirectional [4]. In the last few years, the International Diabetes Federation (IDF) has taken oral health into consideration for DM management [5]. Moreover, clinicians and researchers from medicine and dentistry were brought together at the New York Academy of Sciences conference in 2011, which provided an excellent opportunity and setting for interaction and education to improve the awareness and collaboration across disciplines [6].

DM screening has been considered as a part of routine practice for dentists. With proper medical history obtained from each patient in dental settings, dentists can decrease the chance of events and avoid any complications during the dental treatment visit. History taking can be conducted for various purposes, including detection of possible medical issues, which means that dentists can be considered an important resource of integrated health care providers. Screening for medical conditions in a dental setting is an approach that could be a practical component of a disease prevention/control strategy that integrates health professionals across disciplines.

Globally, it was estimated that in 2017 approximately 462 million individuals were affected by type 2 DM, corresponding to 6.28% of the world’s population [7]. Additionally, the data from the Thai National Health Examination Survey reported the overall prevalence of DM in Thai adults aged ≥ 20 years to be 9.9%, with a higher prevalence among women (10.8%) than men (8.9%) [8]. The prevalence of undiagnosed and diagnosed DM was 4.1% and 5.8%, respectively. The adjusted odds ratio of diabetes prevalence was higher among participants with primary education levels than those with university education levels. The odds of undiagnosed diabetes were higher in the younger age group in both sexes and among women in rural areas compared to urban areas [8]. In our previous study, the prevalence of undiagnosed hyperglycemia and selected associated factors were assessed in a group of Thai dental patients using a point-of-care HbA_1c_. A total of 724 participants were included, and 33.8% had hyperglycemia defined as HbA_1c_ ≥ 5.7%. Older age, family history of DM, being overweight (BMI ≥ 23 kg/m^2^), central obesity, and severe periodontitis were significantly associated with hyperglycemia [9]. Therefore, it was suggested that a dental setting could be an appropriate screening venue for type 2 DM. Oral healthcare professionals could perform an effective type 2 DM screening in dental settings. A study on Thai patients’ attitudes toward screening of DM in dental clinics has been conducted [10]. A total of 601 completed questionnaires were collected; 394 from university/hospital-based dental clinics and 207 from 2 private clinics and a dental faculty’s special (after office hour) clinic. More than 75% of respondents agreed with DM screening in dental clinics. The majority of respondents supported the screening of DM in dental settings, and they were willing to have a screening test by the dentist.

The next logical questions are whether dentists think it is important for them to conduct a chairside screening for DM and whether they would be willing and ready to participate in such activity during the dental visit. Therefore, the objective of this study was to assess the attitudes of dentists toward chairside screening for DM in dental clinics.

## 2. Materials and Methods

This study was approved by the Institutional Review Board of the Faculty of Medicine, Chulalongkorn University (Reference Number: 255/2014), the Committee on Human Right to Human Experimentation of the Faculty of Dentistry/Faculty of Pharmacy, Mahidol University (MU- DT/PY-IRB 2014/057.2611), the Ethics Committee of the Maharat Nakhon Ratchasima Hospital and the Ethics Committee of the Chiangrai Prachanukroh Hospital.

Dentists registered under the Thai Dental Council with at least one year of experience and active practice were included in this study. Dentists who were not currently providing any dental care were excluded. Based on the formula of Taro Yamane [11] at a confidence interval of 95 percent and an acceptance error of 0.05, a minimum of 387 dentists was required for the study. Since a low response rate was anticipated according to a study by Greenberg et al. [12], 60% of this number was added. As a result, a sample size of 620 participants was required to represent a population size of 11,607 dentists who were currently working in Thailand [13].

The survey was performed using two methods: paper-based and online questionnaires. Simple random sampling was utilized to select the regions of Thailand to send the questionnaires. For the paper-based survey, 500 questionnaires were sent to research assistants in each region of Thailand, including North, Northeast, Central, South, and Bangkok, to distribute and collect back from participating dentists. For the online method, the survey was performed using an online questionnaire via Google Forms, an online survey platform operated by Google LLC. A total of 800 questionnaires were sent out via the online method to research assistants who are dentists working in different hospitals throughout the selected regions. The survey was voluntary. Therefore, only the survey data from those willing to answer were sent directly to us.

The questionnaire was newly developed to investigate Thai dentists’ attitudes toward DM screening in a dental setting. First, the content validity of the questionnaire was evaluated by three selected experts: one medical doctor (endocrinologist) and two dentists (community dentistry specialists). The experts agreed upon all questions with the Index of Item Objective Congruence ≥ 0.5 [14]. Next, the questionnaire was tested among 10 dentists with academic and dental backgrounds similar to the targeted participants to evaluate the accuracy and clarity of each question. The internal consistency of our questionnaire was relatively high, with the Cronbach’s alpha coefficients of 0.71.

The final version of the questionnaire consisted of 3 parts. The first part was on the dentist’s demography and the duration of dental practice. The second part evaluated the dentist’s attitude toward DM screening in a dental clinic, which was assessed using the 5-point Likert scale. The scale ranged from 1 being the most negative (i.e., very unimportant, very unwilling, or strongly disagree) to 5 being the most positive answer (i.e., very important, very willing, or strongly agree). Finally, the third part assessed the management of DM on dental patients, which was also evaluated using the 5-point Likert scale. Again, the scale ranged from 1 for the most negative (never perform) to 5 for the most positive answer (always perform).

For the statistical analysis, we analyzed in 3 separated parts according to the questionnaire: the demographic data, the attitudes of the dentists, and the dentist management. All parts were analyzed based on the frequencies and percentages of answers for each question to demonstrate an overview of the survey data. In addition, in the part of the attitudes of the dentists, we assessed the participants’ attitudes by calculating the mean score from the 1–5 response scale and defined the favorable outcome as a response scale of 4 to 5. Finally, we divided the participants according to their specialties. Since periodontists and oral surgeons had to perform surgical interventions, we categorized participants into the general practitioners (GP), periodontists and oral surgeons (POS), and other specialties (OS) groups. The Pearson’s chi-square was used to evaluate the correlation of the favorable score of each question among specialties at the significance level of 0.05. All analyses were completed using SPSS, version 18.0 statistical software (SPSS Inc., Chicago, IL, USA).

## 3. Results

The data in this study were gathered from two methods. In the first method, 500 dentists were invited to participate using paper-based questionnaires distributed via postal mail, and 194 (39%) answered back. An online questionnaire was sent to 800 dentists in the second method, and 438 (54.8%) responded. Overall, the response rate from the two methods was 46.9%. There were no statistically significant differences in the data between the two different methods; therefore, the results from both methods were combined.

Overall, 632 questionnaires were analyzed, and the demographic characteristics of the participants are shown in Table 1. Most of the respondents were female aged 23 to 34 years old (72.1%), who had been practicing for less than 10 years (71.0%) and had never been diagnosed with DM (98.1%). More than half of the participants were specialists, and 39.2% were general practitioners. Among 632 participants, 94 (14.9%) were in the POS group. The remaining 290 (45.9%) participants were in the OS groups, including prosthodontists, endodontists, pedodontists, orthodontists, advanced general dentistry specialists, and operative dentistry specialists. The majority of them worked in hospital-based dental clinics (75.3%).

Table 2 shows the data on the attitudes of the dentists toward DM screening in dental clinics represented on the Likert scale. When the number of participants who answered favorable results was considered, more than half of the respondents indicated that it was crucial for dentists to perform screening of DM in their dental patients (86.3%). Among the potential barriers, respondents felt that the most likely barrier was patients’ willingness (86.4%). Most respondents were willing to measure blood pressure (98.1%) and weight and height (82.4%) for their patients, but less than half were willing to collect blood from fingertips (45.4%) or oral fluids for salivary diagnostics (38.8%). Moreover, more than half of the respondents were ready to refer patients with suspected DM for an evaluation by physicians (73.7%) and were able to explain the relationship between DM and oral diseases (59.5%). On the other hand, fewer respondents were ready to give knowledge about DM awareness (44.3%) and could perform DM screening (27.9%). Furthermore, a large number of the respondents perceived that they knew DM screening criteria (67.2%) and risk factors (65.8%), but less than half knew how to perform blood tests for DM screening (45.1%).

We further investigated the hypothesis that the specialties of the dentists might be one of the significant factors relevant to the corresponding answer in our study. The distribution of favorable answers of the attitudes of the dentists toward DM screening in dental clinics according to the participants’ specialties are presented in Table 3. This table shows that the survey results were similar to those in Table 2. We found statistically significant differences among the specialties with regard to their perception of knowing how to assess DM and feeling prepared to address DM with their patients.

In addition, we found that a significantly higher proportion of dentists in the POS group had a better perception of how to assess DM risk factors, perform blood tests for DM screening, and know the criteria for DM diagnosis compared to the GP and the OS groups. Most of the dentists in the POS group also exhibited their willingness to make dental patients aware of DM, provide information about the relationship of DM and oral diseases, and perform DM screening as part of their routine practice more than the other groups.

**Figure 1:**
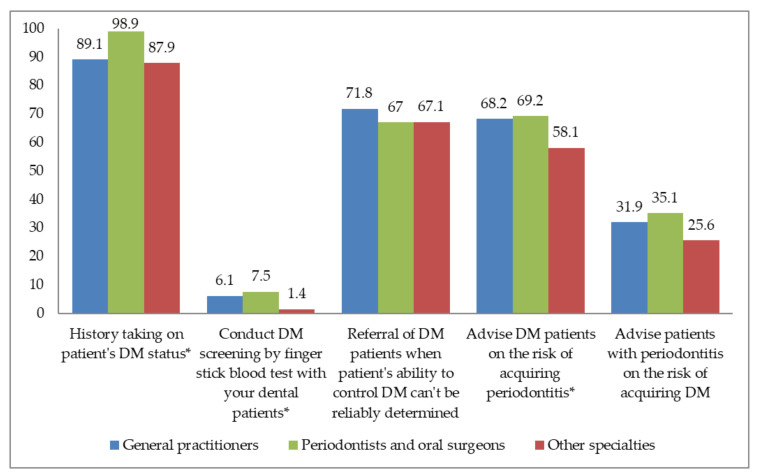
Management of DM screening in dental clinics according to dental specialties. * statistically significant at *p* < 0.05. DM: diabetes mellitus.

Figure 1 presents the data of DM screening in dental clinics according to different dental specialties. An overwhelming majority of respondents from all specialties were likely to ask their patients whether they have DM. Still, less than 10% of the respondents were likely to perform blood tests via fingerstick in patients with DM. A large percentage of respondents referred patients with uncontrolled DM to receive proper medical treatment before starting dental treatment and advised patients with DM about the risk of periodontal diseases. On the other hand, less than half of the dentists whose patients had periodontal disease told their patients about the risk of DM. In general, there was a statistically significant difference between the respondents’ types with respect to inquiring about the patients’ history of DM, conducting DM screening via a fingertip blood sample, and advising the patient with DM about the risk of periodontal disease. However, there was no statistically significant difference between all respondents’ types with respect to referring diabetic patients to receive medical treatment and advising patients with periodontal disease about the risk of DM.

## 4. Discussion

Based on our previous study, Thai patients who attended the dental clinics were willing to have DM screening by their dentists [10]. In addition, another study also reported a positive attitude of Thai physicians toward the screening of DM in dental clinics [15]. Therefore, in the current study, the attitudes of the dentists for the screening of DM were evaluated. We found that 83.6% of dentists agreed that the chairside screening of DM in dental patients was important (Table 2). However, less than one-third (27.9%) reported that they felt prepared to perform chairside screening of DM in their patients (Table 2).

The result from a previous study in the US showed that 76.6% of dentists reported that conducting a DM screening in dental clinics was essential [12]. Another study from California, West Virginia, and Pennsylvania showed that 61% of respondents believed that addressing DM was important to their role as dentists [16]. In addition, giving DM-related advice about periodontal risks and DM-related services depended on the perception of DM management to the dentist’s role. Those who believed their role was important in addressing diabetes were more likely to provide advice and services than those who did not believe. Collectively, these data indicated that most dental patients and dentists realized the importance of DM screening regardless of whether they were in developed or developing countries [16].

Additional data on perceived barriers for dentists to conduct DM screening in dental clinics showed that patients’ willingness was the most important factor. However, data from our previous study suggested that patients were agreeable to participate in the chairside setting of DM screening by dentists [10]. Interestingly, the dentists’ ability to conduct a chairside DM screening was reported as the second most common cause of potential barriers. This indicated that oral healthcare providers require more intensive medical knowledge on DM to implement DM screening in dental clinics successfully. Our findings are comparable to the study of Greenberg et al., which found that patients’ willingness was the greatest potential barrier for dentists to screen for DM in dental settings [17].

Our study showed that the majority of dentists were willing to perform measurements of blood pressure, height, and weight (Table 2). In contrast, only about half of the dentists were ready to collect blood samples, and around 40% of dentists were willing to collect oral fluid samples (Table 2). Low levels of willingness might reflect the concern of difficulty in performing fingerstick blood glucose tests in the dental setting, especially when the dentists are required to have the equipment available in their offices. A previous study in the US similarly reported that about half of the dentists (56%) indicated their willingness to provide fingerstick tests for their patients. [12]. Moreover, the level of acceptance of the dentists to offer fingerstick blood tests in New Zealand was less than that of the US, with a 50% favorable response rate in the group of dentists who recently graduated and only a 20% favorable response rate from dentists who had been graduated for more than 20 years [18].

Our study found that more than 70% of dentists felt prepared to refer dental patients for further medical investigation and treatment (Table 2). On the contrary, less than half of respondents felt prepared to provide DM awareness to their dental patients, and about one-third felt prepared for conducting chairside DM screening (Table 2). A similar result by Esmeili et al. reported that less than half of dental practitioners could evaluate DM patients in dental clinics, and only about 30% were confident in addressing DM with their patients [16]. Moreover, it was found that there was a direct association between training related to DM assessment and management and the practitioners’ confidence in managing DM patients [16].

In the present study, we found that a high percentage of dentists frequently performed DM-related activities such as inquiring dental patients about DM, referring uncontrolled diabetic patients to physicians, and advising diabetic patients about the consequences of uncontrolled DM on periodontal status. However, less than half of all respondents regularly advised their patients with periodontitis about the risk of DM, and less than 10% reported that they usually conducted DM screening by fingerstick blood tests (Figure 1). While practicing dentists appeared to be willing to perform the screening process, very few did. Kunzel et al. reported that only 14% of dentists in general practice either monitored patients’ blood glucose levels or referred them for monitoring [19]. Another study also showed that only 3% of dentists had ever screened their patients for DM using a fingerstick blood test [18]. Esmeili et al. similarly reported that even though 61% of the responding dentists believed that addressing DM was important in their role as a dentist, less than 2% actually performed in-office blood glucose monitoring on diabetic patients [16]. Therefore, while positive attitudes could be found in dentists for DM screening, these attitudes did not translate into the actual clinical screening.

We believed that two major issues impeded the screening for DM in clinical settings. The first one was the availability of a glucometer. Barasch et al. reported that less than 2% of dental offices had glucometer available on-site [20]. In our study, we did not assess the availability of glucometers in clinical settings. However, the survey of glucometer usage suggested that a glucometer was not seen as part of the standard clinical practice of dental patients with DM [20]. Instead, the glucometer was perceived as another domain beyond the scope and responsibility of dentistry. The second issue was the resistance toward screening for medical conditions in general. Whereas monitoring was seen as part of safe and appropriate dental treatment, DM screening was considered a general health service and not a specific screening in dentistry. We believed that using a glucometer to screen undiagnosed DM and hyperglycemia in the dental setting is beneficial. Our previous study indicated that many patients with undiagnosed hyperglycemia and potentially type 2 DM could be identified [21]. Using a glucometer to screen dental patients, the prevalence of hyperglycemia (defined as random blood glucose (RBG) ≥ 110 mg/dL and potentially undiagnosed type 2 DM (defined as RBG ≥ 200) were 63% and 7%, respectively. The sensitivities of using RBG to identify patients with hyperglycemia and potentially undiagnosed type 2 DM were 85.1% and 83.3%, respectively [21]. Therefore, if a glucometer is available in the dental setting and the dentists know how to use it, the identification rate of patients with hyperglycemia or potentially undiagnosed type 2 DM will improve, translating into a better dental treatment outcome for this group of patients.

Currently, DM is a major public health concern worldwide, and the incidence of DM and prediabetes continues to rise. Data in Thailand indicate that at least one-third of DM cases were still undiagnosed; therefore, adding a dental setting as another resource to aid in the case-finding could serve as a potentially helpful global strategy. Screening for chronic medical conditions, such as DM and hypertension in dental clinics, was an approach that received growing interest. Understanding patients’ and dentists’ willingness to participate in medical screening in the dental setting was critical to proceed this strategy forward.

Dentists are uniquely positioned to conduct targeted screening and identify prediabetes patients and those in the early stages of DM; therefore, dental patients are likely to benefit from primary disease prevention strategies. Many studies have supported the notion that primary preventive activities, including dietary modification and increased time spent in physical activities, could reduce the incidence of DM [22,23,24]. DM is associated with a variety of oral complications. Thus, the responsibility falls directly on dental practitioners as they have an opportunity to educate dental patients with DM about these oral complications and, therefore, promote proper oral health behaviors which could reduce the risks of tooth loss, periodontal disease, and oral soft tissue pathologies. Given the professional relationship between patients and dentists, discussion on the screening test results combined with a physician’s follow-up care might be more impactful than screening tests conducted in other non-professional settings, such as health fairs, supermarkets, or pharmacies.

The limitation of this study was its reliance upon self-reported data. Another possible limitation of the data retrieved was the response bias. Individuals who agreed to answer the questionnaires were more likely to have strong opinions in one direction. Another important limitation was the response rate, which was less than 50%. This suggested that the attitudes of most dentists who declined the survey were not captured and not presented. Furthermore, the number of participants in each age group was not equally distributed. Therefore, if a future study is to be conducted, more questionnaires should be sent out, and the age stratification should be considered so that participants in each age group will be more or less equally distributed.

The results from our previous study on patients’ and physicians’ attitudes toward type 2 DM screening and the present study regarding dentists’ attitudes toward type 2 DM screening in the dental office strongly suggested that most of the patients and dentists were aware and had positive attitudes about the importance of screening of DM in the dental office. However, further educational suggestions are necessary, particularly on how to implement such screening and how to improve dentists’ ability to perform DM screening.

## 5. Conclusions

In summary, most dentists consider it essential to conduct DM screening in dental settings. However, they were not sure whether they were ready for the screening. The concerns were difficulties in performing the tests in the office, equipment availability, and their knowledge of DM screening.

While most dentists were ready to refer the patients for further treatment, less than half were ready to provide knowledge about DM awareness to their patients. Furthermore, only one-third of the respondents felt prepared to conduct a chairside DM screening. Therefore, knowledge about DM screening is essential and might be needed for Thai dentists to perform a proper DM screening in dental clinics. In addition, further studies are necessary to determine an appropriate method or process for DM screening in dental settings and to overcome the barriers regarding dentists’ ability to perform DM screening.

## Figures and Tables

**Table 1:** General characteristics of dentists.

Characteristics	Total (*n* = 632)	
	*n*	(%)
**Age (years** **) (*n* = 630)**		
23–34	454	(72.1)
35–44	126	(20.0)
45–54	37	(5.9)
≥55	13	(2.1)
**Gender**		
Male	172	(27.2)
Female	460	(72.8)
**Affiliation**		
Hospital-based	476	(75.3)
Private practice	156	(24.7)
**Specialty**		
None (general practitioners)	248	(39.2)
Periodontists and oral surgeons	94	(14.9)
Other Specialists	290	(45.9)
**Previously diagnosed with DM**		
No	620	(98.1)
Yes	12	(1.9)
**Years in practice**		
≤1	449	(71.0)
>10	183	(29.0)

DM: diabetes mellitus.

**Table 2:** Dentists’ attitudes toward DM screening in dental clinics.

**Topics**	**Total**	**Very** **unimportant**	**Somewhat** **unimportant**	**Not Sure**	**Somewhat** **important**	**Very** **important**	**Favorable score**	**Mean score**
		*n* (%) (1)	*n* (%) (2)	*n* (%) (3)	*n* (%) (4)	*n* (%) (5)	*n* (%) (4 and 5)	
1. How important do you think it is for dentists to conduct chairside DM screening in dental patients?								
	631	4 (0.6)	25 (4.0)	57 (9.0)	322 (51.0)	223 (35.3)	545 (86.3)	4.2
2. If you were considering conducting chairside DM screening in your practice, how important would each of the following issues be?								
2.1. Patients’ willingness	631	7 (1.1)	19 (3.0)	60 (9.5)	289 (45.8)	256 (40.6)	545 (86.4)	4.2
2.2. Additional cost required to perform screening	631	15 (2.4)	89 (14.1)	164 (26.0)	246 (39.0)	117 (18.5)	363 (57.5)	3.6
2.3. Screening Time	631	9 (1.4)	79 (12.5)	90 (14.3)	333 (52.8)	120 (19.0)	453 (71.8)	3.8
2.4. Dentist’s skill to conduct DM screening	631	4 (0.6)	21 (3.3)	80 (12.7)	314 (49.8)	212 (33.6)	526 (83.4)	4.1
**Topics**	**Total**	**Very** **unwilling**	**Somewhat** **unwilling**	**Not sure**	**Somewhat willing**	**Very** **willing**	**Favorable score**	**Mean score**
		n (%) (1)	n (%) (2)	n (%) (3)	n (%) (4)	n (%) (5)	n (%) (4 and 5)	
3. How willing would you be to gather the following samples or data as part of your practice?								
3.1. Blood pressure measurement	628	2 (0.3)	3 (0.5)	7 (1.1)	125 (19.9)	491 (78.2)	616 (98.1)	4.8
3.2. Oral fluids for salivary diagnostic sample	630	43 (6.8)	81 (12.9)	262 (41.6)	161 (25.6)	83 (13.2)	244 (38.8)	3.3
3.3. Height and weight measurements	630	16 (2.5)	32 (5.1)	63 (10.0)	195 (31.0)	324 (51.4)	519 (82.4)	4.2
3.4. Blood via fingerstick	630	65 (10.3)	82 (13.0)	197 (31.3)	193 (30.6)	93 (14.8)	286 (45.4)	3.3
**Topics**	**Total**	**Strongly** **disagree**	**Disagree**	**Not sure**	**Agree**	**Strongly agree**	**Favorable score**	**Mean score**
		n (%) (1)	n (%) (2)	n (%) (3)	n (%) (4)	n (%) (5)	n (%) (4 and 5)	
4. Dentist’s perception of knowing how to assess DM								
4.1. DM risk factors	631	3 (0.5)	55 (8.7)	158 (25.0)	391 (62.0)	24 (3.8)	415 (65.8)	3.6
4.2. Blood tests for DM screening	631	30 (4.8)	101 (16.0)	215 (34.1)	269 (42.6)	16 (2.5)	285 (45.1)	3.2
4.3 DM diagnostic criteria	631	6 (1.0)	54 (8.6)	147 (23.3)	386 (61.2)	38 (6.0)	424 (67.2)	3.6
**Topics**	**Total**	**Very** **unwilling**	**Somewhat** **unwilling**	**Not sure**	**Somewhat willing**	**Very** **willing**	**Favorable score**	**Mean score**
		n (%) (1)	n (%) (2)	n (%) (3)	n (%) (4)	n (%) (5)	n (%) (4 and 5)	
5. How willing would you be to perform the following activities as part of your practice?								
5.1. Educate dental patients about DM awareness	631	5 (0.8)	55 (8.7)	292 (46.3)	237 (37.6)	42 (6.7)	279 (44.3)	3.4
5.2. Explain the relationship between DM and oral diseases	630	4 (0.6)	34 (5.4)	217 (34.4)	295 (46.8)	80 (12.7)	375 (59.5)	3.7
5.3. Screen for DM	631	23 (3.7)	105 (16.6)	327 (51.8)	158 (25.0)	18 (2.9)	176 (27.9)	3.1
5.4. Write a medical referral letter for further investigation and treatment	631	9 (1.4)	25 (4.0)	132 (20.9)	311 (49.3)	154 (24.4)	465 (73.7)	3.9

DM: diabetes mellitus.

**Table 3:** Distribution of favorable answers of the attitudes of the dentists toward DM screening in dental clinics based on specialties.

Topics	Specialty Group			
	General Practitioners	Other Specialists	Periodontists and Oral Surgeons	*p*-Value
	(*n* = 248) n (%)	(*n* = 290) n (%)	(*n* = 94) n (%)	
1. How important do you think it is for dentists to conduct chairside DM screening in dental patients?	215 (86.7)	215 (86.7)	77 (81.9)	0.378
2. If you were considering incorporating DM screening into your practice, how important would each of the following issues be?				
2.1. Patients’ willingness	211 (85.1)	250 (86.5)	84 (89.4)	0.586
2.2. Additional cost required to perform screening	137 (55.2)	172 (59.5)	54 (57.5)	0.607
2.3. Screening time	168 (67.7)	214 (74.1)	71 (75.5)	0.184
2.4. Dentist’s skill to conduct DM screening	210 (84.7)	241 (83.4)	75 (79.8)	0.556
3. How willing would you be to gather the following samples or data as part of your practice				
3.1. Blood pressure measurement	243 (98.4)	282 (97.9)	91 (97.9)	0.911
3.2. Oral fluids for salivary diagnostic	96 (38.9)	116 (40.1)	32 (34.0)	0.573
3.3. Height and weight measurements	210 (85.0)	233 (80.6)	76 (80.9)	0.377
3.4. Blood via fingerstick	125 (50.6)	118 (40.8)	43 (45.7)	0.077
4. Dentist’s perception of knowing how to assess DM				
4.1. DM risk factors	175 (70.6)	166 (57.4)	74 (78.7)	<0.001 *
4.2. Blood tests for DM screening	117 (47.2)	104 (36.0)	64 (68.1)	<0.001 *
4.3. DM diagnostic criteria	171 (69.0)	174 (60.2)	79 (84.0)	<0.001 *
5. How willing would you be to perform the following activities as part of your practice?				
5.1. Educate dental patients about DM awareness	104 (41.9)	119 (41.2)	56 (59.6)	0.005 *
5.2. Explain the relationship between DM and oral diseases	159 (64.4)	152 (52.6)	64 (68.1)	0.004 *
5.3. Screen for DM	73 (29.4)	63 (21.8)	40 (42.6)	<0.001 *
5.4. Write a medical referral letter for further investigation and treatment	180 (72.6)	208 (72.0)	77 (81.9)	0.144

* Statistically significant at *p* < 0.05. DM: diabetes mellitus.

## Data Availability

The data presented in this study are available on request from the corresponding author. The data are not publicly available due to ethical issue.
